# Positive Effect of Probiotics on Constipation in Children: A Systematic Review and Meta-Analysis of Six Randomized Controlled Trials

**DOI:** 10.3389/fcimb.2017.00153

**Published:** 2017-04-28

**Authors:** Ruixue Huang, Jianan Hu

**Affiliations:** Department of Occupational and Environmental Health, Xiangya School of Public Health, Central South UniversityChangsha, China

**Keywords:** probiotics, constipation, children, meta-analysis, randomized controlled trial

## Abstract

**Context:** Constipation in children is a prevalent, burdensome, and psychologically important pediatric issue, the treatment of which remains a global challenge. The use of probiotics has been reported for management of the gastrointestinal microbiota.

**Objective:** This study reviewed the existing literatures of 6 Randomized Control Trials (RCTs) to ascertain some baseline understanding and available information for the effects of probiotics on stool frequency and consistency in children with constipation.

**Data Sources:** PubMed, Springer, Elsevier Science, Cochrane Library, Scopus, Ovid (Medline, EMBASE, PsycINFO), Orbis, and Web of Science from the earliest record in each database to 15 September, 2016.

**Study selection:** Eligible studies were randomized controlled trials that compared the effect of probiotics interventions to any control intervention on stool frequency and consistency.

**Data Extraction:** Studies were identified by searching electronic databases. The meta-analysis was performed by Review Manager 5.3 software using a randomized model.

**Results:** Six studies were identified. The use of probiotics significantly increased the stool frequency [mean difference (MD), 0.73; 95% confidence interval (CI), 0.14–1.31; *P* = 0.02]. Subgroup assessment showed a significantly increased stool frequency in Asian patients (MD, 1.18; 95% CI, 0.33–2.02; *P* = 0.006), but no significant difference in stool consistency (MD, −0.07; 95% CI, −0.21–0.06; *P* = 0.27).

**Limitations:** Only six RCTs met the criteria and were included. Each RCT in this study was performed in a different country, and some of the included studies had a small sample size, which might have influenced the reliability and validity of the conclusions.

**Conclusion:** The present study shows that probiotics increase stool frequency and have beneficial effects in Asian children. However, caution is needed when interpreting these outcomes because of the existence of heterogeneity. Evidence from larger samples and more adequately powered RCTs with results obtained by standardized measurements are necessary to determine which species and dosage of probiotics and what length of treatment are most efficacious for constipation in children.

## Introduction

Constipation, which is characterized by infrequent and painful evacuation, abdominal pain, and fecal incontinence, is a frustrating issue in pediatric healthcare worldwide. The estimated prevalence ranges from 0.7 to 29.6% globally, including both developed and developing countries (Rajindrajith et al., 2016). Constipation is also a familiar disease in pediatric emergency clinics and can have distressing physical effects on affected children, as well as psychological effects on both children and their families (Koppen et al., 2016a). Moreover, constipation is the primary complaint in 3–5% of children who present to pediatric physicians and represents the cause of 10–25% of transfers to special gastroenterologists (Molnar et al., 1983). Liem (Liem et al., 2009) estimated that the cost associated with childhood constipation in the United States is ~$3430/per case/per year.

According to the recommendations of the North American Society for Pediatric Gastroenterology, Hepatology, and Nutrition, treatment of childhood constipation usually includes parental or family education, dietary changes, toilet training, use of medications such as laxatives, and behavioral modification (Constipation Guideline Committee of the North American Society for Pediatric Gastroenterology, Hepatology and Nutrition, 2006). Although these methods are popular, the majority of children require sustained therapy for a long period of time, and some children with constipation do not achieve satisfactory results. Statistical data show that even when treatment is sustained for 1 year, about 50% of children remain symptomatic and about 30% still struggle with this disorder (Poddar, 2016). Therefore, interest in the development and evaluation of new and more effective solutions for constipation in children is growing.

Technological developments have shown that the gut microbiota is essential for human health and that a wide variety of childhood diseases are associated with the condition of the gut microbiota (Johnson and Versalovic, 2012). Probiotics are attracting increasing attention in this regard. They are defined by the World Health Organization as live microorganisms that, when taken in certain amounts, lead to health benefits for the host (Quigley, 2015). That means probiotics are live bacteria and yeasts which has a expand spectrum including the well-known strains of *Lactobacillus acidophilus, Bifidobacterium lactis*, and *Lactobacillus brevis* that are good for your health, not only the digestive system but also other systems such as brain. In general in terms of bacteria we think it can lead to diseases, however, probiotics are typically called “good” or “helpful” bacteria because they help keep healthy. A large amount of evidence is available on the benefits of using probiotics as a strategic therapy for various gastrointestinal disorders including persistent diarrhea, community-acquired acute diarrhea, and irritable bowel syndrome (Bernaola Aponte et al., 2010; Applegate et al., 2013; Quigley, 2015; Szajewska et al., 2016). Probiotics also reportedly have potential for the treatment of constipation in children. In 2005, Benninga et al. stated that probiotics represent a new treatment option for childhood constipation (Benninga et al., 2005). Bekkali et al. (2007) conducted a pilot study to determine the effect of a mixture of probiotics including *Bifidobacterium* and *Lactobacillus* spp. on constipation and indicated that these probiotics had positive effects on the symptoms of constipation; for instance, they increased the frequency of bowel movements and decreased the number of fecal incontinence episodes per week in children (Bekkali et al., 2007). A study by Bu et al. (2007) demonstrated no significant difference in treatment efficacy between a probiotics group (*Lactobacillus casei rhamnosus*, Lcr35) and a control group of children with chronic constipation. Khodadad (Khodadad and Sabbaghian, 2010) used synbiotics to treat childhood constipation and found that it improved symptoms of childhood constipation without any side effects. In addition, Guerra et al. (2011) used *Bifidobacterium*-containing yogurt to examine its effects on childhood chronic constipation and found that the defecation frequency and severity of abdominal pain were improved. Furthermore, in 2011, a double-blind randomized controlled trial (RCT) conducted by Tabbers et al. (2011b) showed that a fermented dairy product containing *B. lactis* strain DN-173010 increased the stool frequency with no serious side effects. In the same year, these authors also found that *Bifidobacterium breve* was effective in increasing the stool frequency in children with functional constipation (Tabbers et al., 2011c). Chen (Chen et al., 2012) conducted a prospective cohort study in Taiwan in 2012 and found that mothers with higher education levels, families with higher incomes, and parents with a healthier lifestyle tended to use more probiotic supplementation for their children. These findings indicate that probiotics have a much more positive influence in this population. Moreover, in 2013, a study by Saneian et al. (2013) revealed that supplementation of mineral oil with the synbiotic Lactol (containing *L. sporogenes*) can improve the constipation symptoms of children without side effects. In 2014, Sadeghzadeh et al. also investigated the effectiveness of probiotics in the treatment of constipation in children and found that the stool frequency increased and the stool consistency improved with probiotics (Sadeghzadeh et al. 2014). However, according to a study by Banaszkiewica (Banaszkiewicz and Szajewska, 2005), *Lactobacillus* GG was not an effective adjunct to lactulose in treating constipation in children.

The human microbiome is a topic of interest for many researchers and may alter our views of health and disorders in the next several decades. To the best of our knowledge, however, the role of the human microbiome in treating constipation in children is unclear because of contradictory research findings and the lack of systematic reviews and meta-analyses of the efficacy of probiotics for constipation in children. Thus, we conducted a systematic review of RCTs to summarize the evidence of the relationship between probiotics and constipation in children and to identify heterogeneity among the RCT findings.

## Methods

### Inclusion criteria

The inclusion criteria were as follows: (1) The study evaluated children aged ≤ 18 years with constipation identified by relative clinical symptoms, pediatric physicians, or the Rome I, II, or III criteria. (2) The study design was an RCT. (3) Any type of culture/strain/dose/therapy regimen of probiotics was included. Synbiotics were also included because they consist of both prebiotics and probiotics (Koppen et al., 2016b). Any medication form including tablet, powder, oil suspension, or capsule was included. (4) The study included a clinical cohort and controls, and the clinical cohort's intervention was the consumption of probiotics. (5) Clinical studies used similar methods and measured stool frequency and stool consistency. The results were reported as mean ± standard deviation. When the same groups of patients were reported in multiple papers, only the most recent and complete paper was selected to avoid overlap.

### Exclusion criteria

Studies that met the following criteria were excluded from the meta-analysis: (1) those with populations of patients aged >18 years with constipation; (2) pilot studies, cross-sectional studies, or other investigations without a randomized control group; (3) outcomes were presented in other ways such as figures without numerical outcomes of mean ± standard deviation; and (4) not meeting the inclusion criteria described above.

### Article search strategy

Two independent researchers performed searches of the electronic databases of PubMed, Springer, Elsevier Science, Cochrane Library, Scopus, Ovid (Medline, EMBASE, PsycINFO), Orbis, and Web of Science from the earliest record in each database to 15 September, 2016. The following key words were used: “probiotics,” “prebiotics,” “synbiotics,” “*Lactobacillus*,” “*Bifidobacterium*,” “*Saccharomyces*,” “childhood constipation,” “constipation in children,” and “randomized controlled trials.” We also checked the references listed at the end of each publication to identify additional studies; however, only studies published in the English language were considered, and those comprising only conference abstracts were excluded due to the lack of sufficient data.

### Data collection

Data collection was performed by two reviewers independently. Any eligible studies with regard to the effect of probiotics on constipation in children were collected on a tailored form and examined by the second reviewer. The form included study's author(s), publication date, population demographics, probiotic species, probiotic dosage, and treatment results (Table 1). If the study data were unclear, such as the standard deviation was lacking, we attempted to contact the corresponding author to obtain further information in detail.

**Table 1:** **Characteristics of included RCTs for meta-analysis**.

**Author, year (country)**	***N***	**Age**	**Constipation definition**	**Genus, species, and strain**	**Dose**	**Duration (weeks)**
Banaszkiewicz and Szajewska, 2005 (Poland)	84	Probiotics: 79 months Placebo: 65 months	<3 BMs per week for at least 12 weeks	*Lactobacillus rhamnosus GG*	10^9^colony-forming units, twice daily orally	12
Bu et al., 2007 (Taiwan)	45	Probiotics: 36.7 months Placebo: 35 months	Having a stool frequency of <3 times per week for >2 months	*Lactobacillus casei rhamnosus*, Lcr35	8 × 10^8^c.f.u., two capsules, b.i.d	4
Khodadad et al., 2010 (Iran)	102	Synbiotics + liquid paraffin: 5.9 years, Synbiotics + Placebo: 6.2 years, Liquidparaffin + placebo: 6.9 years	Rome III criteria	*L. casei, L. rhamnosus, S. thermophilus, B. breve, L.acidophilus, B. infantis*	1 × 10^9^CFU/1 sachet, per day	4
Sadeghzadeh et al., 2014 (Iran)	48	Probiotics: 6.1 years Control: 6.3 years	Rome III criteria	*Lactobacillus casei, Lactobacillus rhamnosus PXN54, Streptococcus thermophiles PXN66, Bifidobacterium breve PXN25, Lactobacillus acidophilus PXN35, Bifidobacterium infantis PXN27, Lactobacillus bulgaricus*	1 × 10^9^CFU	4
Saneian, 2013, (Iran)	60	Probiotics: 5.4 years Control: 4.7 years	Rome III Criteria	*Lactobacillus Sporogenes*	15 × 10^7^ spores,1 Tab/20 kg/day	8
Tabbers, 2011, (Netherlands, Poland)	159	Probiotics: 7 years Control: 6.5 years	Rome III criteria	*Lactobacillus delbruec kiissp. Bulgaricus CNCM strain numbers I-1632 and I-1519, Streptococcus the rmophilus CNCM strain, Lactococcuscremoris Blactis DN-173 010*	1.2 × 10^8^CFU per pot, two pots per day	3

### Statistical analysis

The meta-analysis was carried out with RevMan 5.3 software (Cochrane Collaboration 2014, Nordic Cochrane Center, Copenhagen, Denmark). Stool frequency and stool consistency were the major variables used to verify the efficacy of probiotics in the treatment of constipation in children. Because the results were continuous outcomes, the mean difference (MD) and 95% confidence interval (CI) were calculated for the summary statistics analysis, and a randomized-effects model was utilized based on the heterogeneity of the results among the studies. Subgroup analyses were also conducted for different geographic areas and different bacterial strains.

In the meta-analysis, heterogeneity across studies was assessed by the I (Koppen et al., 2016a) statistic. Statistical heterogeneity was audited using the χ^2^-test, and the extent of inconsistency was assessed by the *I*^2^ statistic. If *I*^2^ ≥ 50%, which indicated significant heterogeneity, we used a random-effects model for the analysis; otherwise, we used a fixed-effects model to assess the variables. Publication bias was assessed by the funnel plot. A two-tailed *p* < 0.05 was considered statistically significant. We performed the sensitivity analysis by excluding the studies one by one. Using the Cochrane “risk of risk” assessment tool, we assessed the risk of bias for each included RCT.

## Results

### Included studies

**Figure 1:**
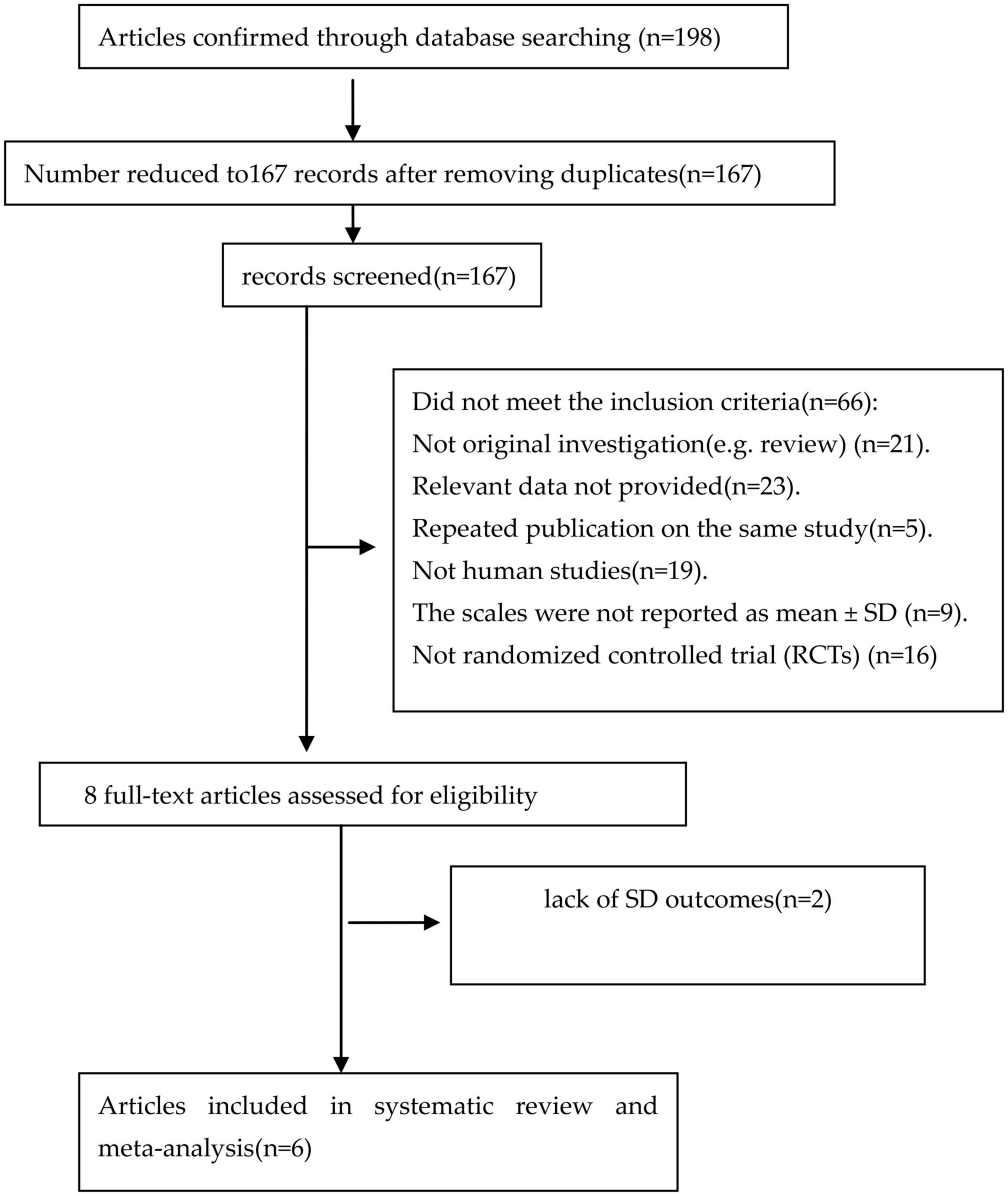
**PRISMA flow diagram of studies included in meta-analysis**.

An adapted PRISMA flow diagram was used to present the process of article selection for the meta-analysis (Hutton et al., 2016). Figure 1 shows the flow diagram of studies enrolled in the meta-analysis. In the initial search, 198 articles were reviewed. At the end of the flow diagram, six studies involving 498 children met the selection criteria (Banaszkiewicz and Szajewska, 2005; Bu et al., 2007; Khodadad and Sabbaghian, 2010; Tabbers et al., 2011b; Saneian et al., 2013; Sadeghzadeh et al., 2014). The characteristics of the studies are shown in Table 1.

### Quality assessment

**Figure 2:**
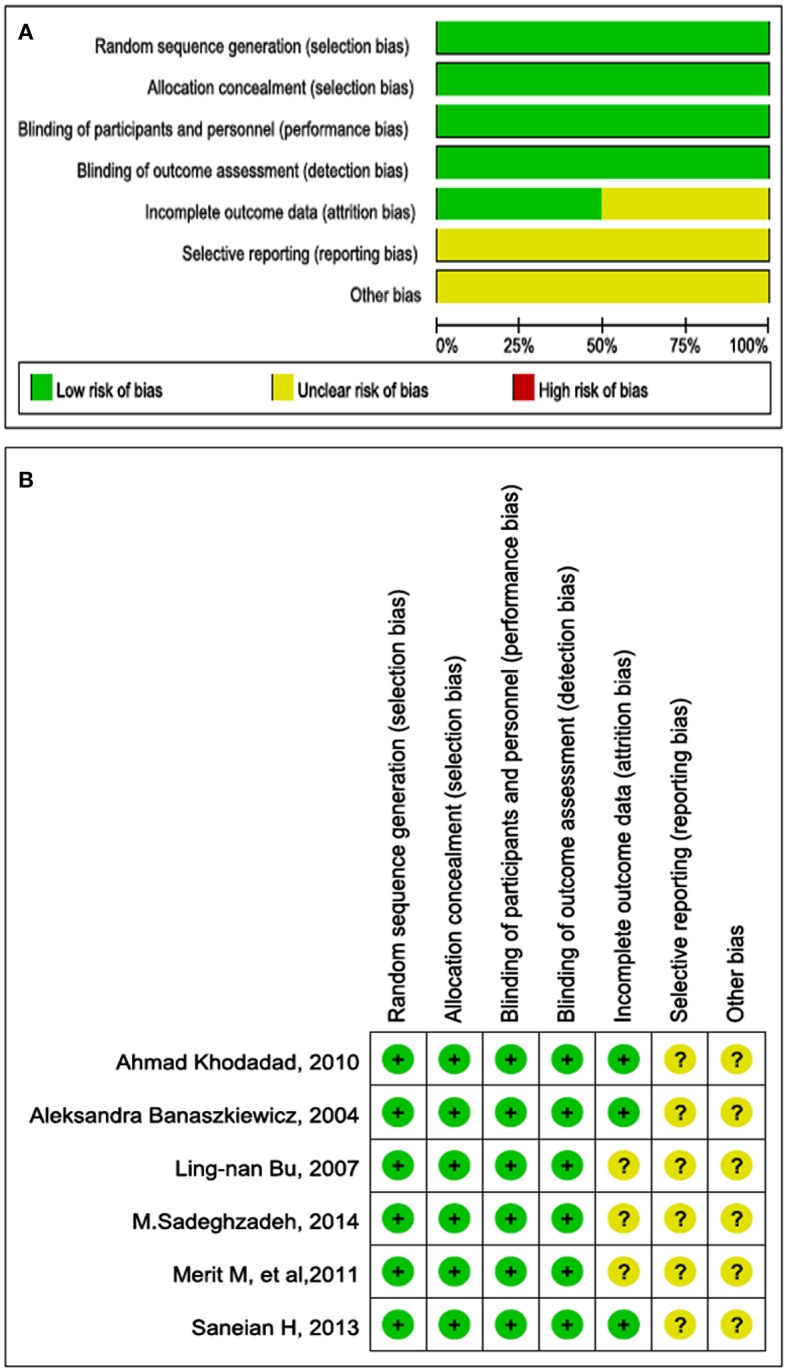
**(A)** Bar chart comparing percentage risk of bias for each included RCT. The risk of bias was quite low. **(B)** Risk of bias for each included RCT, representing low risk of bias (+), high risk of bias (−), and unclear risk of bias (?).

Figure 2A shows the risk of bias across all RCTs. A plot of the distribution of the review authors' judgments regarding the risk-of-bias items across all studies is shown. Figure 2B presents a summary table of the review authors' judgments regarding each risk-of-bias item for each study. All six studies were RCTs, and risk of bias for each included RCT was low. None of the studies had a high risk of bias. Four studies divided the children into a probiotics intervention group and a control group; two studies randomly divided the children into three groups: probiotics, placebo, and other intervention. Five of the six studies were double-blind studies. All six studies reported the baseline data of each group, and the differences in these data among the groups were not statistically significant in all studies.

### Efficacy of probiotics stool frequency

**Figure 3:**
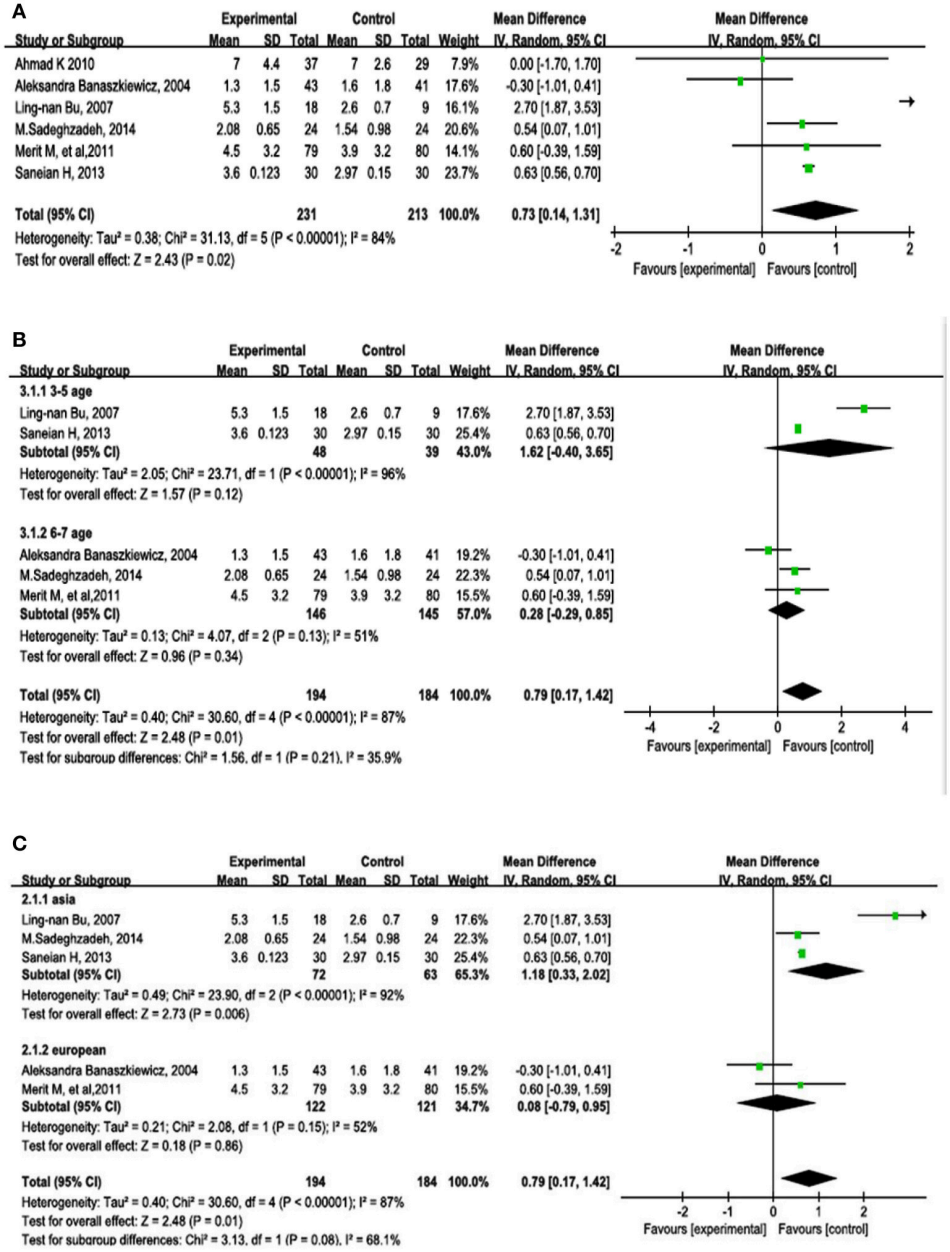
**Forest plots of RCTs in patients with constipation comparing probiotics with placebo or other intervention**. Random-effects models were used to analyze the MD and 95% CI. **(A)** Overall outcomes of the six studies. **(B)** Subgroup analysis by age. **(C)** Subgroup analysis by geographical area.

Stool frequency was measured in all six studies. The results are shown in Figure 3. The random-effects model comparing the probiotics and control groups showed an MD of 0.73 (95% CI, 0.14–1.31; *P* = 0.02).

A subgroup analysis was carried out to determine whether age affected the outcome of treatment by probiotics (Figure 3B). The ages of the children in two of the studies ranged from 3 to 5 years (MD, 1.62; 95% CI, −0.4–3.65; *P* = 0.12). The ages of the children in two other studies ranged from 6 to 7 years (MD, 0.28; 95% CI, −0.29–0.85; *P* = 0.34).

A subgroup analysis was also performed to determine whether geographical areas affected the outcome of treatment by probiotics (Figure 3C). Four studies were from Asia (MD, 1.18; 95% CI, 0.33–2.02; *P* = 0.006) and two studies were from Europe (MD, 0.08; 95% CI, −0.79–0.95; *P* = 0.86).

The stability of the results was tested by sensitivity analysis. We sequentially removed studies that did not reach statistical significance in all of the above analyses, suggesting that the results of our meta-analysis were not significantly unstable.

#### Stool consistency

**Figure 4:**
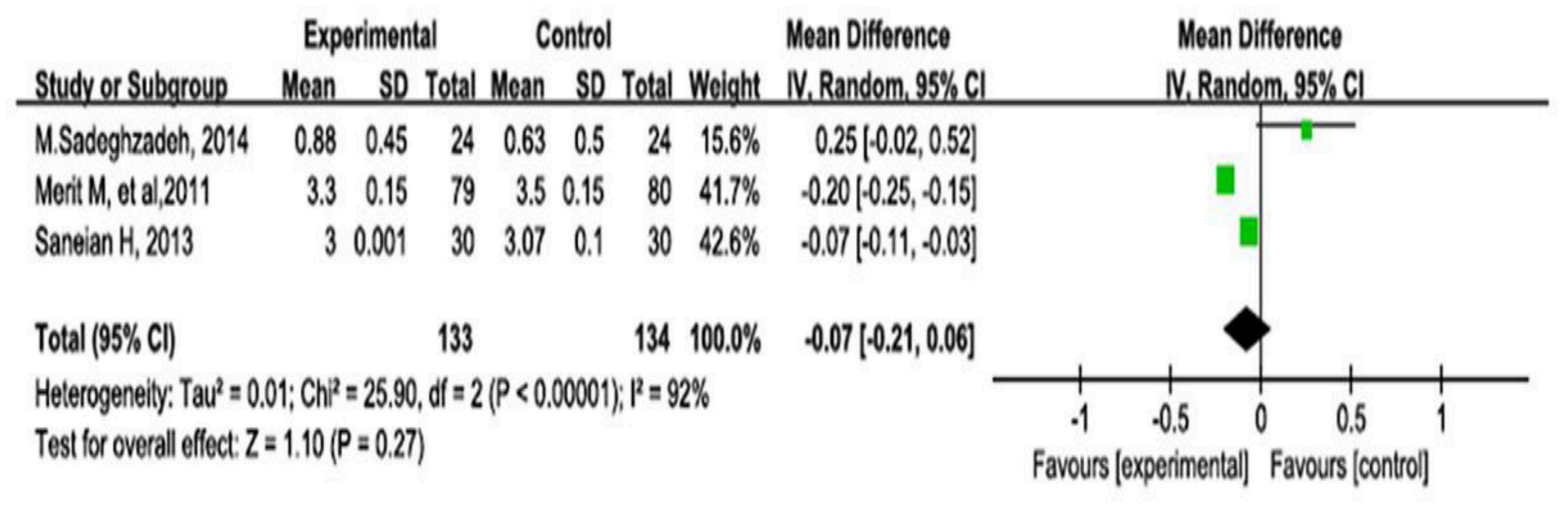
**Forest plot of outcome of meta-analysis of RCTs regarding stool consistency in children with constipation**.

Stool consistency was measured in three studies; the remaining three studies did not report the outcomes of stool consistency. As shown in Figure 4, the meta-analysis using a random-effects model comparing the probiotics and control groups showed an MD of −0.07 (95% CI, −0.21–0.06; *P* = 0.27).

### Publication bias

**Figure 5:**
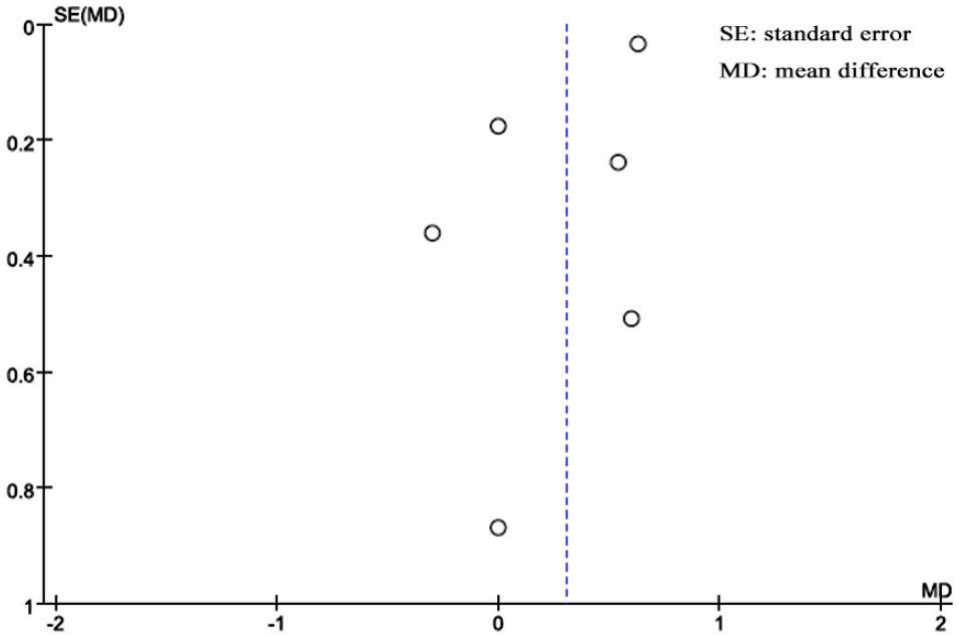
**Funnel plot of publication bias analysis**.

A funnel plot was used to assess publication bias qualitatively. The funnel plot shown in Figure 5 is partially symmetrical, indicating no obvious evidence of asymmetry and therefore no evidence of publication bias.

## Discussion

Various studies have investigated the potential mechanisms of action by which probiotics might play a role in the treatment of constipation in children. One proposed mechanism is that probiotics beneficially change the gastrointestinal microbiota (Grehan et al., 2010). Another is that some probiotics have an antinociceptive role in which they inhibit contraction of the colonic epithelial cell cytoskeleton, which opens the tight junctions, and induce direct or indirect effects of nitric oxide in the gastrointestinal tract (Ait-Belgnaoui et al., 2006). A third proposed mechanism is that some probiotics increase the amounts of lactate and short-chain fatty acids as well as decrease the luminal pH, thus enhancing gut peristalsis (Waller et al., 2011). We performed the present meta-analysis based on the hypothesis that probiotics significantly increase stool frequency and modify stool consistency. In our previous study, we reported that probiotics had a significant effect on decreasing the depression scale score (Huang et al., 2016). In the present study, we continued to assess the effect of probiotics on other disorders such as constipation, which is common among children. The outcomes of this study demonstrate that probiotics result in a significantly increased stool frequency (Figure 3); however, there was significant heterogeneity (*I*^2^ = 80%, *P* = 0.02). We conducted subgroup assessments and found different effects of probiotics on stool frequency in children depending on geographical area; Asian children had a significantly higher stool frequency with probiotic treatment.

Stool frequency is a critical variable with which to assess the treatment of constipation (Tabbers et al., 2011a). The present study showed heterogeneity in the outcome of stool frequency, which positively increased by probiotic supplementation; this was particularly pronounced in Asian children. A meta-analysis on the effects of probiotics on constipation in adults published in 2014 showed outcomes similar to those of the present study (Dimidi et al., 2014). Our assessment in children and other findings in adults show that probiotics have a significant role in changing stool frequency in both children and adults. It was reported that probiotics, especially some most studied strains such as *Lactobacilli* and *Bifidobacteria* are able to produce short chain fatty acids reducing the level of intraluminal pH and promoting colonic peristalsis, which is beneficial for changing stool frequency. We carried out a subgroup assessment of age; however, the findings showed no significant difference between the 3- to 5-year-old group and the 6- to 7-year-old group. The reason for this finding may have involved the number of patients enrolled. Larger study samples have the potential to improve the statistical outcomes.

According to the Rome III criteria, stool consistency is another important index with which to evaluate constipation (Koppen et al., 2016c). Khodadad et al. (Khodadad and Sabbaghian, 2010) studied the effects of *L. casei, L. rhamnosus, Streptococcus thermophilus, B. breve*, and other microorganisms on stool consistency and found no statistically significant difference. In addition, Tabbers et al. (2011b) used fermented milk containing *B. lactis* DN-173010 to treat constipation in children, and the outcomes showed that stool consistency was not significantly different between the probiotic group and the control group. We also found that probiotics had no significant effect on improving stool consistency in children. Our findings are similar to those of former reports.

This meta-analysis contributes clinically important information on the treatment of constipation in children. When pediatric physicians treat constipation in children, probiotics represent an alternative strategy that has been shown to be efficacious, especially in terms of increasing stool frequency, which is an important measurement in constipation. On the other hand, parents often treat their children with over-the-counter medications or laxatives, which might not relieve the symptoms (Borowitz and Ritterband, 2001). Probiotics are an alternative approach for parents when their children have this problem.

We made many efforts to minimize publication bias in this meta-analysis; however, despite comprehensive and complete document retrieval and performance of the analysis by two separate researchers, this study has some limitations. First, heterogeneity of some findings was significant, which means that there was variation among the studies. This could have been caused by differences in the types and doses of probiotics among the studies, as well as differences in other factors such as diet, body condition, other medications, and sample size. Second, each RCT in this study was performed in a different country; thus, people with different genetic constitutions or microbial exposure may have had different responses to identical probiotics. Third, some of the included studies had a small sample size, which might have influenced the reliability and validity of the conclusions.

## Conclusion

This meta-analysis provides some baseline understanding and available information that probiotics have the potential to increase the stool frequency; however, the findings must be interpreted with caution because of heterogeneity. Regardless, the outcomes are a source of optimism with respect to the management of constipation in children. Further evidence from larger samples and more adequately powered RCTs that use standardized measurements are necessary to assess which species and dosage of probiotics and what length of treatment are most efficacious for constipation in children.

## Author contributions

JH: conceived and designed the study, performed eligibility screening, and did data extraction. After initial manuscript was completed, he critically revised the manuscript and polished the English language. He approved the final manuscript as submitted. RH: conceived and designed the study, performed eligibility screening and data extraction; as well as analyzed the data and performed the statistical analysis. She wrote the initial manuscript. He approved the final manuscript as submitted. All authors approved the final manuscript as submitted and agree to be accountable for all aspects of the work.

### Conflict of interest statement

The authors declare that the research was conducted in the absence of any commercial or financial relationships that could be construed as a potential conflict of interest.

## Abbreviations

RCTs: randomized controlled trials
MD: mean difference.

## References

[B1] Ait-Belgnaoui A. Han W. Lamine F. Eutamene H. Fioramonti J. Bueno L.. (2006). *Lactobacillus farciminis* treatment suppresses stress induced visceral hypersensitivity: a possible action through interaction with epithelial cell cytoskeleton contraction. Gut 55, 1090–1094. 10.1136/gut.2005.084194 16507583 PMC1856261

[B2] Applegate J. A. Fischer Walker C. L. Ambikapathi R. Black R. E. (2013). Systematic review of probiotics for the treatment of community-acquired acute diarrhea in children. BMC Public Health 13(Suppl. 3):S16. 10.1186/1471-2458-13-S3-S16 24564646 PMC3847198

[B3] Banaszkiewicz A. Szajewska H. (2005). Ineffectiveness of Lactobacillus GG as an adjunct to lactulose for the treatment of constipation in children: a double-blind, placebo-controlled randomized trial. J. Pediatr. 146, 364–369. 10.1016/j.jpeds.2004.10.022 15756221

[B4] Bekkali N. L. Bongers M. E. Van den Berg M. M. Liem O. Benninga M. A. (2007). The role of a probiotics mixture in the treatment of childhood constipation: a pilot study. Nutr. J. 6:17. 10.1186/1475-2891-6-17 17683583 PMC2148043

[B5] Benninga M. A. Candy D. C. Taminiau J. A. (2005). New treatment options in childhood constipation? J. Pediatr. Gastroenterol. Nutr. 41(Suppl. 1), S56–S57. 10.1097/01.scs.0000180307.02052.56 16131972

[B6] Bernaola Aponte G. Bada Mancilla C. A. Carreazo Pariasca N. Y. Rojas Galarza R. A. (2010). Probiotics for treating persistent diarrhoea in children. Cochrane Database Syst. Rev. CD007401. 10.1002/14651858.CD007401.pub2 23963712 PMC6532736

[B7] Borowitz S. M. Ritterband L. (2001). Using the internet to teach parents and children about constipation and encopresis. Med. Inform. Internet Med. 26, 283–295. 10.1080/14639230110086295 11783712

[B8] Bu L. N. Chang M. H. Ni Y. H. Chen H. L. Cheng C. C. (2007). *Lactobacillus casei* rhamnosus Lcr35 in children with chronic constipation. Pediatr. Int. 49, 485–490. 10.1111/j.1442-200X.2007.02397.x 17587273

[B9] Chen Y. C. Chien Y. W. Chang P. J. Hsieh W. S. Chen P. C. (2012). Probiotic supplement use among young children in Taiwan: a prospective cohort study. PLoS ONE 7:e43885. 10.1371/journal.pone.0043885 22984450 PMC3440429

[B10] Constipation Guideline Committee of the North American Society for Pediatric Gastroenterology, Hepatology and Nutrition. (2006). Evaluation and treatment of constipation in infants and children: recommendations of the North American Society for Pediatric Gastroenterology, Hepatology and Nutrition. J. Pediatr. Gastroenterol. Nutr. 43, e1–13. 10.1097/01.mpg.0000233159.97667.c3 16954945

[B11] Dimidi E. Christodoulides S. Fragkos K. C. Scott S. M. Whelan K. (2014). The effect of probiotics on functional constipation in adults: a systematic review and meta-analysis of randomized controlled trials. Am. J. Clin. Nutr. 100, 1075–1084. 10.3945/ajcn.114.089151 25099542

[B12] Grehan M. J. Borody T. J. Leis S. M. Campbell J. Mitchell H. Wettstein A. (2010). Durable alteration of the colonic microbiota by the administration of donor fecal flora. J. Clin. Gastroenterol. 44, 551–561. 10.1097/MCG.0b013e3181e5d06b 20716985

[B13] Guerra P. V. Lima L. N. Souza T. C. Mazochi V. Penna F. J. Silva A. M.. (2011). Pediatric functional constipation treatment with Bifidobacterium-containing yogurt: a crossover, double-blind, controlled trial. World J. Gastroenterol. 17, 3916–3921. 10.3748/wjg.v17.i34.3916 22025880 PMC3198021

[B14] Huang R. Wang K. Hu J. (2016). Effect of prbiotics on depression: a systematic review and meta-analysis of randomized controlled trials. Nutrients 8:E483. 10.3390/nu8080483 27509521 PMC4997396

[B15] Hutton B. Catalá-López F. Moher D. (2016). [The PRISMA statement extension for systematic reviews incorporating network meta-analysis: PRISMA-NMA]. Med. Clin. 147, 262–266. 10.1016/j.medcli.2016.02.025 27040178

[B16] Johnson C. L. Versalovic J. (2012). The human microbiome and its potential importance to pediatrics. Pediatrics 129, 950–960. 10.1542/peds.2011-2736 22473366 PMC3340594

[B17] Khodadad A. Sabbaghian M. (2010). Role of synbiotics in the treatment of childhood constipation: a double-blind randomized placebo controlled trial. Iran. J. Pediatr. 20, 387–392. 23056736 PMC3446081

[B18] Koppen I. J. Benninga M. A. Tabbers M. M. (2016b). Is there a role for Pre-, Pro- and synbiotics in the treatment of functional constipation in children? A systematic review. J. Pediatr. Gastroenterol. Nutr. 63(Suppl. 1), S27–S35. 10.1097/MPG.0000000000001220 27380596

[B19] Koppen I. J. Di Lorenzo C. Saps M. Dinning P. G. Yacob D. Levitt M. A.. (2016a). Childhood constipation: finally something is moving! Expert Rev. Gastroenterol. Hepatol. 10, 141–155. 10.1586/17474124.2016.1098533 26466201

[B20] Koppen I. J. Velasco-Benitez C. A. Benninga M. A. Di Lorenzo C. Saps M. (2016c). Using the bristol stool scale and parental report of stool consistency as part of the Rome III criteria for functional constipation in infants and toddlers. J. Pediatr. 177, 44–48. 10.1016/j.jpeds.2016.06.055 27453373

[B21] Liem O. Harman J. Benninga M. Kelleher K. Mousa H. Di Lorenzo C. (2009). Health utilization and cost impact of childhood constipation in the United States. J. Pediatr. 154, 258–262. 10.1016/j.jpeds.2008.07.060 18822430

[B22] Molnar D. Taitz L. S. Urwin O. M. Wales J. K. (1983). Anorectal manometry results in defecation disorders. Arch. Dis. Child. 58, 257–261. 10.1136/adc.58.4.257 6847228 PMC1627951

[B23] Poddar U. (2016). Approach to constipation in children. Indian Pediatr. 53, 319–327. 10.1007/s13312-016-0845-9 27156546

[B24] Quigley E. M. (2015). Probiotics in irritable bowel syndrome: the science and the evidence. J. Clin. Gastroenterol. 49(Suppl. 1), S60–S64. 10.1097/mcg.0000000000000348 26447967

[B25] Rajindrajith S. Devanarayana N. M. Crispus Perera B. J. Benninga M. A. (2016). Childhood constipation as an emerging public health problem. World J. Gastroenterol. 22, 6864–6875. 10.3748/wjg.v22.i30.6864 27570423 PMC4974585

[B26] Sadeghzadeh M. Rabieefar A. Khoshnevisasl P. Mousavinasab N. Eftekhari K. (2014). The effect of probiotics on childhood constipation: a randomized controlled double blind clinical trial. Int. J. Pediatr. 2014:937212. 10.1155/2014/937212 24812563 PMC4000641

[B27] Saneian H. Tavakkol K. Adhamian P. Gholamrezaei A. (2013). Comparison of *Lactobacillus sporogenes* plus mineral oil and mineral oil alone in the treatment of childhood functional constipation. J. Res. Med. Sci. 18, 85–88. 23914206 PMC3724383

[B28] Szajewska H. Canani R. B. Guarino A. Hojsak I. Indrio F. Kolacek S.. (2016). Probiotics for the prevention of antibiotic-associated diarrhea in children. J. Pediatr. Gastroenterol. Nutr. 62, 495–506. 10.1097/MPG.0000000000001081 26756877

[B29] Tabbers M. M. Boluyt N. Berger M. Y. Benninga M. A. (2011a). Nonpharmacologic treatments for childhood constipation: systematic review. Pediatrics 128, 753–761. 10.1542/peds.2011-0179 21949142

[B30] Tabbers M. M. Chmielewska A. Roseboom M. G. Crastes N. Perrin C. Reitsma J. B.. (2011b). Fermented milk containing *Bifidobacterium lactis* DN-173 010 in childhood constipation: a randomized, double-blind, controlled trial. Pediatrics 127, e1392–e1399. 10.1542/peds.2010-2590 21606153

[B31] Tabbers M. M. de Milliano I. Roseboom M. G. Benninga M. A. (2011c). Is *Bifidobacterium breve* effective in the treatment of childhood constipation? Results from a pilot study. Nutr. J. 10:19. 10.1186/1475-2891-10-19 21345213 PMC3048518

[B32] Waller P. A. Gopal P. K. Leyer G. J. Ouwehand A. C. Reifer C. Stewart M. E.. (2011). Dose-response effect of *Bifidobacterium lactis* HN019 on whole gut transit time and functional gastrointestinal symptoms in adults. Scand. J. Gastroenterol. 46, 1057–1064. 10.3109/00365521.2011.584895 21663486 PMC3171707

